# Magnetic resonance mammography in the evaluation of recurrence at the prior lumpectomy site after conservative surgery and radiotherapy

**DOI:** 10.1186/bcr1600

**Published:** 2006-09-07

**Authors:** Lorenzo Preda, Gaetano Villa, Stefania Rizzo, Luca Bazzi, Daniela Origgi, Enrico Cassano, Massimo Bellomi

**Affiliations:** 1 Department of Radiology, European Institute of Oncology IRCCS, 435 Via Ripamonti, 20141 Milan, Italy; 2 School of Medicine, University of Milan, Via di Rudini 8, 20133 Milan, Italy; 3 Department of Medical Physics, European Institute of Oncology IRCCS, 435 Via Ripamonti, 20141 Milan, Italy; 4 Breast Imaging Unit, Department of Radiology, European Institute of Oncology IRCCS, 435 Via Ripamonti, 20141 Milan, Italy

## Abstract

**Introduction:**

The aim was to assess the value of magnetic resonance mammography (MRM) in the detection of recurrent breast cancer on the prior lumpectomy site in patients with previous conservative surgery and radiotherapy.

**Methods:**

Between April 1999 and July 2003, 93 consecutive patients with breast cancer treated with conservative surgery and radiotherapy underwent MRM, when a malignant lesion on the site of lumpectomy was suspected by ultrasound and/or mammography. MRM scans were evaluated by morphological and dynamic characteristics. MRM diagnosis was compared with histology or with a 36-month imaging follow-up. Enhancing areas independent of the prior lumpectomy site, incidentally detected during the MRM, were also evaluated.

**Results:**

MRM findings were compared with histology in 29 patients and with a 36-month follow-up in 64 patients. MRM showed 90% sensitivity, 91.6% specificity, 56.3% positive predictive value and 98.7% negative predictive value for detection of recurrence on the surgical scar. MRM detected 13 lesions remote from the scar. The overall sensitivity, specificity, positive predictive value and negative predictive value of MRM for detection of breast malignancy were 93.8%, 90%, 62.5% and 98.8%, respectively.

**Conclusion:**

MRM is a sensitive method to differentiate recurrence from post-treatment changes at the prior lumpectomy site after conservative surgery and radiation therapy. The high negative predictive value of this technique can avoid unnecessary biopsies or surgical treatments.

## Introduction

Recurrence of breast cancer lesions on the surgical scar after conservative surgery and radiation therapy have been reported to occur in at least 1–2% of cases per year [1,2]. The proper follow-up of these patients usually includes periodic clinical examination, mammography and ultrasonography [3]. Detection of recurrence on the prior lumpectomy site still represents a challenge because of changes in breast tissue after treatment. Clinical examination, mammography or ultrasonography can raise a suspicion but an additional evaluation is frequently mandatory to avoid unnecessary biopsy or surgery.

Several recent studies have shown the important role of breast magnetic resonance imaging (MRI) imaging for detection of recurrent lesions in patients treated with conservative surgery (quadrantectomy) and radiation therapy [3-5]. Magnetic resonance mammography (MRM) has high sensitivity, high specificity and high accuracy in differentiating physiologic changes of the scar from tumoral tissue [3,6-10]. MRM multifactorial evaluation, based on both morphological features and time–signal intensity curves of enhancing lesions, is related to significantly higher sensitivity and higher specificity than evaluation protocols based only on one morphological or enhancing feature [1].

To the best of our knowledge, no specific data are available on the accuracy of MRM to differentiate recurrent lesions from normal changes of the surgical scar at the site of prior lumpectomy when a multifactorial MRM evaluation protocol is used to classify enhancing lesions.

This study was designed to determine whether MRM, performed with a multifactorial evaluation of enhancing lesions, improves the accuracy of diagnosis of recurrence on the surgical scar in patients who underwent quadrantectomy and local radiation therapy. Furthermore, the overall accuracy of contrast-enhanced MRM for the detection of suspicious enhancing lesions, even when not closely related to the surgical scar, has also been assessed in the series reported.

## Materials and methods

### Patients

The study cohort comprised 93 female patients who underwent breast MRI examination between April 1999 and July 2003 for suspected recurrence on the site of conservative surgery (quadrantectomy) for breast cancer, at least 6 months after the end of radiation therapy.

All patients underwent a yearly breast evaluation, performed by the breast radiologist with mammography, associated or not with an ultrasound examination, according to the characteristics of the breast tissue density, and underwent a clinical examination. The clinical examination routinely performed by the breast radiologists after the evaluation of mammographic scans, matched in the same report of mammography, and was also used to decide the level of suspicion.

Recurrence was suspected either by ultrasound or by mammography, or by both examinations. When a malignant lesion on the surgical scar was suspected (ultrasound and/or mammographic Breast Imaging Reporting and Data System (BI-RADS) III or BI-RADS IV), the patient underwent a contrast-enhanced breast magnetic resonance mammography (MRM), after signing a proper informed consent and after exclusion of contraindications to exposure to magnetic fields.

### Magnetic resonance mammography technique

All MRM examinations were performed on a Signa Horizon LX (GE Medical Systems, Milwaukee, WI, USA), with a field strength of 1 T. The entire breast was scanned with a two-channel, phased-array, bilateral dedicated coil and the following parameters: Repetition Time = 7 ms, Echo Time = 1.6 ms, flip angle = 10°, TI = 25 ms, receiver bandwidth = 32 kHz, Number of Excitations = 1, matrix = 320 × 320, Field of View = 36 × 18 cm^2^. The slice thickness was chosen between 2 mm and 3 mm, depending on the breast size, in order to maintain each sequence time within 90 s.

Coronal T1-weighted, fat-suppressed (spectral inversion at lipid) FSPGR 3D sequences were acquired once before and five times after intravenous contrast injection (Gadopentetate dimeglumine, 0.2 mmol/kg; flow rate = 2 ml/s). The acquisition of dynamic images started 10 s after the contrast injection.

To determine the contrast medium uptake, baseline images were subtracted from images obtained after contrast medium injection. All dynamic images were sent to a diagnostic workstation (Advantage Window 4.2; GE Medical Systems, Milwaukee, WI) where the maximum intensity projection and the multiplanar reformation were obtained.

### Evaluation of magnetic resonance images and classification of lesions

One senior radiologist, aware of the time of surgery and the location of the primary breast cancer, and of the mammographic and ultrasonographic findings, reviewed each MRM examination on a diagnostic workstation.

Each lesion was retrospectively reviewed and classified by the reader according to a multifactorial evaluation protocol for enhancing lesions connected or not to the surgical scar. Morphological features and time–signal intensity curves of enhancing lesions were classified according to the Fischer multifactorial evaluation [1] to differentiate malignant lesions and benign lesions.

The morphological features evaluated and graded were form, margins and enhanced pattern. Form was considered suspicious for malignancy when it was branching or spiculated, whereas it was considered benign when it was rounded. Margins were considered suspicious for malignancy if indistinct, while they were considered benign if well defined. The enhancing pattern was suspicious for malignancy if it was inhomogeneous or ring shaped, whereas it was considered benign if it was homogeneous. Lesions were accordingly graded on a one-point scale for form and for margins (0 = benign, 1 = malignant), and on a two-point scale for enhancing patterns (0 = benign, 1 = suspicious, 2 = malignant).

According to the same classification of Fischer and colleagues [1], the dynamic course of enhancement was considered suspicious for malignancy if there was an initial strong increase and a postinitial washout, while a moderate initial increase and a postinitial plateau were considered benign. Accordingly, a two-point scoring scale system was used both for the initial increase of enhancement (0 = enhancement <50%, 1 = 50–100% enhancement, 2 = enhancement >100%) and for the postinitial enhancement (0 = steady increase, 1 = plateau, 2 = washout).

Using this multifactorial evaluation protocol, the overall maximum score achievable is 8: a total score lower than 3 points indicated benign lesions, whereas a total score higher than 4 points indicated malignant lesions.

The American College of Radiology recently introduced a classification for lesions detected by MRM (adapted from the mammographic BI-RADS classification) [11] in five classes: BI-RADS I = negative finding, BI-RADS II = benign finding, BI-RADS III = probably benign finding, BI-RADS IV = suspicious finding, and BI-RADS V = a finding highly suggestive of malignancy.

The Fischer score was accordingly converted into the MRM-BI-RADS score of the American College of Radiology, as follows: Fischer 0, 1 = BI-RADS I; Fischer 2 = BI-RADS II; Fischer 3 = BI-RADS III; Fischer 4 and Fischer 5 = BI-RADS IV; Fischer 6, Fischer 7 and Fischer 8 = BI-RADS V.

MRI findings were compared either with histological findings, if the patient underwent biopsy or surgery, or with a 36-month follow-up with the usual imaging modalities (ultrasound and/or mammography), if the patient did not undergo any biopsy or surgery.

### Statistical analysis

True-positive, true-negative, false-positive and false-negative cases were recorded as follows. True-positive cases were patients with histological signs of disease within 3 months after the MRM indicating the lesion as suspicious for recurrence. True-negative cases were patients with negative findings at MRM who had negative findings at ultrasonography and/or mammography during the following 36-month follow-up. False-positive cases were patients who underwent biopsy or surgery because MRM indicated an enhancing lesion as suspicious for malignancy but histological findings and follow-up examinations were negative for malignancies. False-negative cases were patients who underwent biopsy or surgery within 3 months after a negative MRM, because of clinical, ultrasound or mammographic findings suspicious for recurrence. These patients were considered false negative at MRM if histological examination demonstrated the presence of cancer.

The sensitivity, specificity, positive predictive value and negative predictive value and the accuracy of MRM in the detection of recurrent disease on the surgical scar were calculated. The same values, with 95% confidence intervals relating to the sensitivity and specificity, were calculated for the lesions detected in areas not related to the scar, in order to define the overall accuracy of MRM in the detection of new breast cancer lesions.

## Results

Ninety-three patients (mean age, 53.3 years; range, 40–72 years) were evaluated in the present study: 40 patients had conservative surgery on the right breast, and 53 patients had conservative surgery on the left breast. Follow-up evaluation after surgery included mammography for all 93 patients and ultrasound for 74 patients. Recurrence was suspected by mammography in 27 patients (15 patients graded as mammographic BI-RADS III, 12 patients graded as BI-RADS IV), by ultrasound in 58 patients (30 patients graded as US BI-RADS III, 28 patients graded as BI-RADS IV), and by both examinations in eight patients (one patient graded as both mammographic and ultrasound BI-RADS III, two patients graded as ultrasound BI-RADS III and mammographic BI-RADS IV, three patients graded as ultrasound BI-RADS IV and mammographic BI-RADS III, two patients graded as both ultrasound and mammographic BI-RADS IV).

The mean time of follow-up after MRM, performed with mammography, ultrasonography or both examinations, was 36 months (range, 12–48 months). The mean age of patients at the time of new diagnosis was 52 years.

MRM findings were confirmed by histological findings in 29 patients and by follow-up imaging modalities (ultrasound and/or mammography) in 64 patients.

The 93 lesions studied by MRM on the surgical scar, evaluated according to the Fischer criteria, included 59 lesions graded as Fischer 0, two lesions graded as Fischer 1, eight lesions graded as Fischer 2, eight lesions graded as Fischer 3, 12 lesions graded as Fischer 4, no lesions graded as Fischer 5, four lesions graded as Fischer 6, and no lesions graded as Fischer 7 and Fischer 8. The same lesions subsequently classified into the magnetic resonance BI-RADS classification were: 61 lesions graded as BI-RADS I, eight lesions graded as BI-RADS II, eight lesions graded as BI-RADS III, 12 lesions graded as BI-RADS IV, and four lesions graded as BI-RADS V (Table 1).

**Figure 1:**
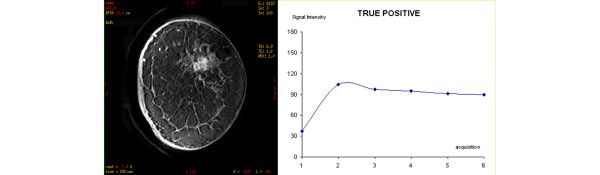
**Magnetic resonance mammography 4 years after treatment for invasive ductal carcinoma. **This shows a 2 cm lesion (left image), with a signal–intensity curve with early wash-in and late washout (right image) (Breast Imaging Reporting and Data System V) (invasive ductal carcinoma at histology).

In the evaluation of enhancing lesions on the surgical scar, nine lesions were true-positive cases. All of these lesions were confirmed positive by histological examination. In these cases, malignancy was suspected by ultrasound alone in five cases (three cases of BI-RADS III, two cases of BI-RADS IV), by mammography alone in two cases (both graded as mammographic BI-RADS IV), and by both examinations in two cases (one case graded as mammographic BI-RADS IV and ultrasound BI-RADS III, one case graded as both mammographic and ultrasound BI-RADS IV). Histological proof of malignancy was obtained with a vacuum-assisted breast biopsy in four patients, with a tru-cut biopsy in three cases, with fine-needle aspiration cytology in one case, and with fine-needle aspiration cytology + tru-cut in one case. Four out of nine patients underwent a complete mastectomy, 2/9 underwent a second conservative treatment (quadrantectomy), 1/9 died without undergoing further surgery (histological confirmation of recurrence came from biopsy), and the remaining 2/9 underwent surgery in other institutions – we received the pathological report without a detailed description of the type of surgery. Histological types of the true-positive cases were 8/9 infiltrating ductal carcinoma and 1/9 cribriform infiltrating carcinoma. The mean dimension of lesions was 1.96 cm (range, 0.6–3 cm). Six out of these nine lesions were graded as Fischer 4, and the other three lesions were graded as Fischer 6 (Figure 1).

There were 76 true-negative cases: 64 patients with a negative follow-up, nine patients with negative histological findings, and three patients with a negative histology and follow-up. Suspicion of malignancy was raised by ultrasound in 47 cases (25 cases graded as ultrasound BI-RADS III, 22 cases graded as ultrasound BI-RADS IV), by mammography in 24 cases (15 cases graded as mammographic BI-RADS III, nine cases graded as mammographic BI-RADS IV), and by both examinations in five cases (one case graded as both ultrasound and mammographic BI-RADS III, one case graded as ultrasound BI-RADS III and mammographic BI-RADS IV, two cases graded as ultrasound BI-RADS IV and mammographic BI-RADS III, one case graded as both ultrasound and mammographic BI-RADS IV). Fifty-nine of these 76 cases were graded as Fischer 0, two lesions were graded as Fischer 1, seven lesions were graded as Fischer 2, and eight lesions were graded as Fischer 3. Among lesions graded as Fischer 3 (probably benign), three cases underwent biopsy while five cases underwent a 6-month MRI and a 12-month MRI as the patients refused to undergo a biopsy.

**Figure 2:**
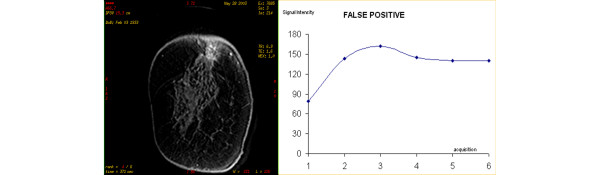
**Magnetic resonance mammography 15 months after surgery. **The postcontrast image (left image) and the signal–intensity curve (right image) show a 2 cm lesion with inhomogeneous enhancement. (Breast Imaging Reporting and Data System IV). Histology and follow-up reported no malignancy.

Seven false-positive cases were detected on the surgical scar based on histological findings. Malignancy was suspected by ultrasound alone in five cases (two cases graded as ultrasound BI-RADS III, three cases graded as ultrasound BI-RADS IV), by mammography alone in one case (graded as BI-RADS IV), and by both examinations in one case (graded as mammographic BI-RADS III and ultrasound BI-RADS IV). Six of these false-positive cases were graded at MRM as Fischer 4 (Figure 2) and one case was graded as Fischer 6. The mean time between primary treatment (surgery and radiotherapy) and MRM was 13 months (range, 6–24 months). Two out of seven patients underwent the MRM less than 12 months after treatment (6 months and 8 months, respectively).

**Figure 3:**
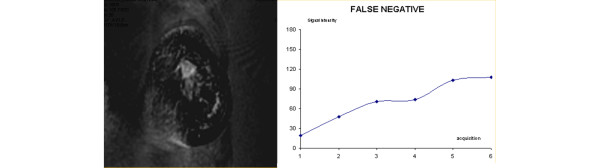
**Magnetic resonance mammography of 52-year-old patient who underwent quadrantectomy, adenectomy and radiotherapy for mucinous cancer. **This shows a 3 cm rounded lesion with homogeneous contrast enhancement (left image), with the signal–intensity curve showing moderate initial increase and late steady increase (right image). The lesion (Breast Imaging Reporting and Data System II) was mucinous cancer.

We found only one false-negative case at MRM. The ultrasonographic detection of a hypoechoic inhomogeneous lesion of 2 cm in correspondence with the surgical scar (ultrasound BI-RADS IV) gave indication to perform MRM. The subsequent MRM scan depicted the lesion as rounded, with regular shape and homogeneous contrast enhancement. The time–intensity curve showed a moderate initial increase and a steady increase in later sequences (Figure 3). The lesion was therefore graded as a Fischer 2 (BI-RADS II) lesion. Despite the negative result of the MRM, the patient underwent surgery after 2 months because of the ultrasonographic suspicion, and a 2 cm mucinous cancer was demonstrated on the surgical scar. The primary malignancy in this patient was again a mucinous type cancer.

Thirteen lesions remote from the surgical scar of prior lumpectomy were detected in seven patients. These lesions were classified as: one Fischer 2, four Fischer 3, three Fischer 4, two Fischer 5, two Fischer 6, and one Fischer 8. The same lesions according to the BI-RADS classes were: 0 cases graded as BI-RADS I, one case graded as BI-RADS II, four cases graded as BI-RADS III, five cases graded as BI-RADS IV, and three cases graded as BI-RADS V.

**Figure 4:**
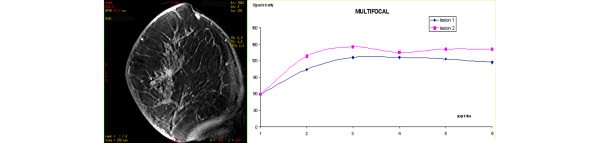
**Magnetic resonance mammography 2 years after therapies for invasive ductal carcinoma. **This shows two lesions (arrows) (Breast Imaging Reporting and Data System IV, V), reported by biopsy as malignant.

Six true-positive cases were identified, one case graded as Fischer 4, two cases graded as Fischer 5, two cases graded as Fischer 6, and one case graded as Fischer 8. Among these patients, 3/6 had no malignancy on the surgical scar: one of them underwent a controlateral quadrantectomy; the remaining two patients, having enhancing lesions in a different quadrant, underwent a total mastectomy. Three out of six patients had second cancer foci distant from the scar as well as on the scar, confirmed by histology as multifocal carcinomas: in two cases the enhancing lesions were on the same quadrant and the patients underwent a second quadrantectomy, and in one case the lesion was in another quadrant and the patient underwent a total mastectomy (Figure 4). For 3/6 true-positive cases, all graded as BI-RADS IV at mammography, second-look ultrasound examinations were performed: in all three cases the lesion was detected. Only in 1/3 had an ultrasound examination been performed before the MRM, and it was considered negative.

Two false positive-cases were detected; they were graded as Fischer 4, demonstrated as negative by histological findings and confirmed by follow-up.

Five true-negative cases confirmed by negative findings during the follow-up were graded as follows: one case graded as Fischer 2, and four cases graded as Fischer 3.

During the follow-up, no enhancing lesions remote from the scar were detected in any of the 80 patients with negative MRM, thus confirming the absence of false-negative results.

The sensitivity, specificity, negative predictive value, positive predictive value and accuracy of MRM for the detection of recurrence on the surgical scar were, respectively, 90%, 91.6%, 98.7%, 56.3% and 91.4%. The overall sensitivity, specificity, negative predictive value, positive predictive value and accuracy of MRM in the detection of breast cancer lesions (including areas not related to the scar) were, respectively, 93.8% (95% confidence interval, 82–100%), 90% (95% confidence interval, 83–96%), 98.8%, 62.5% and 90.6%.

## Discussion

The long-term survival rate among women who undergo breast-conserving surgery is the same as that among women who undergo radical mastectomy [12]. Breast-conserving surgery is therefore currently considered the treatment of choice for women with relatively small breast cancers [12], and the rate of recurrence reported in these patients is about 1–2% per year [1,2]. Long-term survival of patients with new malignancy after conservative treatment improves with early detection [10,13].

The diagnostic evaluation of the treated breast is unfortunately still a challenge because post-treatment changes of breast tissue can show great variability, hiding or mimicking recurrent lesions [3,14]. Changes following breast-conserving surgery can include hematoma, seroma, fat tissue necrosis, scar tissue development and dystrophic calcifications [15]. Changes after radiotherapy can include vascular dilatation, capillary damage, microcirculatory changes and edema [15,16]. The association of these changes after breast-conserving surgery and irradiation make the interpretation of clinical examination and mammography very difficult because of focal thickening, decreased compressibility and increased density at the surgical site [3,13].

At our institution the usual follow-up of breast cancer patients treated with breast-conserving surgery and irradiation is based on annual imaging evaluation performed with mammography, completed by clinical examination and/or ultrasound examination, according to the density of the breast.

Local recurrence is the development of a tumor in the ipsilateral (treated) breast that occurs after treatment of the initial breast cancer [17]. Comparison of studies relating to the detection of recurrence can be difficult because of differences in the definitions of local recurrence [17]. Mammography has limited sensitivity in these lesions [8,18], because of an insufficient morphological distinction between therapy-induced edema and lymphangiosis carcinomatosa or between radially striated scar tissue and tumor recurrence [15]. This lack of specificity can delay early detection and treatment of recurrent lesions [8]. The ultrasound examination is used as a complementary follow-up imaging modality of the treated breast, especially in radiodense breasts. This examination does have high sensitivity if performed at regular intervals, because of the detection of hypoechoic nodules within fibrous hyperechoic tissue [3]. The sensitivity of ultrasound is user dependent, however, and it is limited when evaluating small or noninvasive lesions, especially in fatty breasts.

For these reasons, MRM is considered a useful additional examination in patients with suspicion of recurrence [7,8]. Progress in breast MRI has been limited by a lack of standardization in the acquisition and interpretation of MRM images, with some studies focusing on morphology (spatial resolution) [19] and other studies stressing kinetics (temporal resolution) [20,21].

In the present study, the multifactorial evaluation protocol proposed by Fischer and colleagues [1] was used to evaluate enhancing lesions on the site of prior lumpectomy, based both on their morphologic and kinetic characteristics, in an attempt to standardize their evaluation. The number of true-negative cases confirmed by histology (12 lesions) demonstrates that 12 unnecessary biopsies could have been avoided using this multifactorial protocol.

The dynamic enhancement pattern, combined with morphology, on contrast-enhanced MRI of breast masses, allows reproducible lesion characterization [22], and it is useful to differentiate between benign and malignant lesions [21,23]. A review of the literature has reported a sensitivity of more than 90%, and 85–100% specificity, for MRM to detect recurrence [7,10,24-26]. In the present study MRM correctly detected 8/9 lesions on the surgical scar, all being infiltrating carcinomas.

The sole false-negative lesion was an infiltrating mucinous carcinoma. The lesion showed well-defined margins, regular shape and homogeneous contrast enhancement, with a moderate initial increase and a steady increase in later sequences (graded as Fischer 2 and BI-RADS II). Other cases of infiltrating mucinous breast cancer not detected by MRM have been described [27,28]. The large amount of mucus slows the diffusion of contrast medium through the entire tumor [27,29], and thereby induces gradual enhancement curves [30,31]. T2-weighted images have been considered useful because of the very high signal intensity on these sequences compared with other histologic types [29]. In this case, the T2-weighted sequence would probably have been helpful in detecting the mucinous lesion. Nevertheless, this type of sequence is not routinely performed in our institution because of the low rate of this histological type (1–4%) [29].

MRM in the present study found seven false-positive cases, although all examinations were performed at least 6 months after treatment. Other studies have demonstrated difficulties in differentiating a recurrent tumor from postoperative changes within 6 months after surgery [32] and within 12 months after radiation therapy [33]. In the seven false-positive cases described herein, the mean time between treatments and MRM was 13 months, and only in two of these cases was the time between the end of radiotherapy and MRM shorter than 12 months (6 months and 8 months, respectively).

The high sensitivity in detection of multifocality, in particular for lesions located far from the scar, has influenced the type of repeat surgery (quadrantectomy versus mastectomy). These results are comparable with other studies [9] performed on smaller numbers of patients.

Of particular relevance is the very high negative predictive value of MRM (98.7%), which indicates a very low likelihood of new malignancy if MRM defines the lesion as benign. These results suggest that lesions graded by MRM as Fischer I–II (BI-RADS I–II) can be safely monitored with the usual yearly follow-up. A repeat MRM examination after 6 months is recommended for lesions graded as Fischer III (BI-RADS III), if there is no clinical suspicion of recurrence before 6 months. For lesions graded higher than Fischer IV (BI-RADS IV–V), further cytological or histological evaluation is mandatory.

One limitation of this study is the lack of *in situ *ductal carcinoma in our series. Ductal carcinoma *in situ *is considered one of the most common causes of false-negative results at MRM [34], and this can decrease the diagnostic accuracy of the technique. In the present series we found no ductal carcinoma *in situ*, and this does probably justify the 93.8% overall sensitivity with only one false-negative case. Another limitation was that imaging examinations were evaluated by a single reviewer. We therefore did not assess the interobserver variability in the use of this multifactorial protocol.

## Conclusion

This series demonstrates the high sensitivity and high specificity of MRM in confirming or excluding recurrence at the prior lumpectomy site, after conservative surgery and radiation therapy, when recurrence was already suspected either by mammography (including the associated clinical examination) or by ultrasonography. MRM has shown an overall high negative predictive value (98.8%) in the detection of breast cancer, including lesions not related to the surgical scar.

Despite the high accuracy of MRM in detecting recurrence on the site of lumpectomy, its cost and low availability limit its use for the routine follow-up of treated patients. In some conditions, however, as in the presence of radiodense breasts and/or structural post-treatment changes, MRM represents an important diagnostic modality in support of the other traditional imaging modalities, and it can be considered conclusive when showing negative findings.

## Abbreviations

BI-RADS = Breast Imaging Reporting and Data System; MRI = magnetic resonance imaging; MRM = magnetic resonance mammography.

## Competing interests

The authors declare that they have no competing interests.

## Authors' contributions

LP contributed to the study concept and design, manuscript drafting and editing, approval for important intellectual concepts, and literature research. GV contributed to the clinical study, data acquisition, literature research, and manuscript drafting and editing. SR contributed to the clinical study, data acquisition, statistical analysis, literature research, and manuscript drafting and editing. LB contributed to the clinical study, data acquisition, literature research and manuscript drafting. DO contributed to the clinical study, data acquisition, data analysis and interpretation, literature research, and manuscript drafting and editing. EC contributed to the clinical study, data acquisition, literature research and manuscript drafting. MB contributed to the study concept and design, manuscript drafting and editing, approval for important intellectual concepts, data analysis and interpretation. All authors approved the final version of the submitted manuscript.

## Figures and Tables

**Table 1:** Magnetic resonance mammography findings

Fischer score	BI-RADS score	Diagnostic value	Total lesions (*n*)	True-negative lesions (*n*)	False-negative lesions (*n*)	False-positive lesions (*n*)	True-positive lesions (*n*)
0	I	Negative	59	59			
1	I	Negative	2	2			
2	II	Benign	8	7	1		
3	III	Probably benign	8	8			
4	IV	Suspicious abnormality	12			6	6
5	IV	Suspicious abnormality	0				
6	V	Suggestive of malignancy	4			1	3
7	V	Suggestive of malignancy	0				
8	V	Suggestive of malignancy	0				

Classification of lesions according to the Fischer scores (0–8) and Breast Imaging Reporting and Data System (BI-RADS) scores (I–V), and the corresponding diagnostic value.
